# The Role of FSHR SNPs and AMH in Follicular Fluid and Serum in Ovarian Response during COS: A Pilot Study

**DOI:** 10.1155/2021/8685158

**Published:** 2021-02-09

**Authors:** Elli Anagnostou, Despina Mavrogianni, Ilectra-Niki Prifti, Evangelia Dimitroulia, Athanasios Protopapas, Peter Drakakis, Dimitrios Loutradis

**Affiliations:** 1st Department of Obstetrics and Gynecology, Division of Human Reproduction, IVF Unit, Alexandra Hospital, Medical School of National Kapodistrian University of Athens, Athens, Greece

## Abstract

**Background:**

Several studies have investigated on the polymorphism Ser680Asn of FSHR and its use as a predictive indicator of response to an IVF/ICSI protocol. Furthermore, measurement of AMH in serum and follicular fluid is a useful prognostic indicator for the outcome of an assisted reproduction attempt. The purpose of this study is to examine the FSH receptor Ser680Asn polymorphism in combination with AMH levels in both serum and follicular fluid, on the day of oocyte collection.

**Materials and Methods:**

A total of 32 women who underwent IVF/ICSI were included. Women were grouped into 2 groups: those who received rFSH (*n* = 11) and those who received hMG (*n* = 21). Serum AMH was measured on day 3 of the cycle, and AMH in the follicular fluid on the day of oocyte retrieval; the same day peripheral blood was collected for the genotyping of Ser680Asn.

**Results:**

No statistical significant difference was found between serum AMH and follicular fluid AMH regarding the FSH receptor genotype for the Ser680Asn polymorphism. Regarding the sAMH/ffAMH ratio in the 3 genotypes, the value was lower in Asn/Asn women than Ser/Ser and Ser/Asn, but no statistical difference was obtained. Women who carry the Ser allele have a higher number of follicles, retrieved oocytes, and mature oocytes than women who do not contain the Ser allele. Women with AMH < 2.22 ng/ml presented lower AMH follicular fluid levels and lower serum AMH/follicular fluid AMH ratio in a statistically significant manner. Concerning the genotype for the polymorphism Ser680Asn of FSHR in relation to AMH levels, no statistically significant differences were found.

**Conclusions:**

The identification of polymorphisms, such as Ser680Asn of FSHR, along with the determination of endocrine markers in the follicular fluid, such as AMH, could lead at some point, to the personalized therapy setting per woman.

## 1. Introduction

Genetic polymorphism is defined as the occurrence of more than one allele in a genetic locus within a population, and in addition, each allele must occur at a rate of at least 1% of the population [1].

Mutations and polymorphisms in the FSH receptor (FSHR) gene could affect the reproductive ability in men and especially in women [2, 3]. Of the eight polymorphisms in the coding region of FSHR, two (Thr307Ala and Ser680Asn) have been extensively studied in ART protocols, in order to evaluate the stimulation of FSH receptor by gonadotropins [4]. Genetic variants of FSHR are also subject of study in the evolving field of pharmacogenetics, as a tool to select the most appropriate treatment protocol in individuals undergoing *in vitro* fertilization (IVF) [5, 6]. Several studies have investigated on the polymorphism Ser680Asn of FSHR and its use as a predictive indicator of response to an IVF/ICSI protocol. Knowledge of certain polymorphisms, of the FSHR gene or others, could provide information on their role on ovarian stimulation and on the selection of the appropriate protocol which would ensure a sufficient number of mature oocytes for IVF/ICSI [7].

Towards that direction, Perez Mayorga et al. in 2000 demonstrated that the ovarian response to the stimulation with gonadotropins is associated with polymorphisms of the FSHR gene [8]. They observed that women who were homozygous for the Ser allele (Ser/Ser) needed higher doses of exogenously administered FSH, compared with women carrying the Asn allele (Asn/Asn and Ser/Asn). In 2002, Sudo and colleagues reported a statistically significant difference in serum FSH levels between different genotypes [9]. In 2003, de Castro et al. observed higher proportion IVF cycle cancellations in women who were homozygous for Ser (Ser/Ser), a greater distribution of this genotype in poor responders and lower response rate to stimulation to administered recombinant FSH as well [10]. In contrast to the above, the implantation rate and pregnancy rate as stated in the study of Klinkert in 2006 was higher in women with Ser/Ser genotype with respect to the Asn/Asn [11]. The same year, Loutradis and colleagues reported a significant correlation between the dose of gonadotropins in women who participated in an IVF program and levels of FSH [12]. The fact that women with genotype Ser/Ser have elevated levels of FSH and therefore require higher doses of exogenous FSH during an IVF program was also reported by Yao in 2011 [13].

AMH is a dimeric glycoprotein that belongs to the TGF-*β* family (transforming growth factor-*β*). In humans, the AMH gene is located at chromosome 19 and consists of five exons [14]. AMH is a product of granulosa cells of the preantral and small antral follicles in women. Follicles of size 5-8 mm follicles make the greatest contribution to serum AMH [15]. Subsequently, as the follicles increase in size, AMH production decreases and pauses [16]. Production of AMH regulates folliculogenesis by inhibiting recruitment of follicles from the resting pool in order to select for the dominant follicle, after which the production of AMH diminishes [17, 18].

Several studies have linked AMH levels with the ovarian reserve, the ovarian response of women in IVF protocols, the outcome of IVF attempt, the age of menopause, and other conditions related to woman's physiology and pathology [19–23].

In a study conducted by Hazout et al. in 2004, a significant correlation was found between serum AMH levels and IVF outcome in women less than 42 years old: higher AMH levels were associated with more mature oocytes, more good quality of embryos, and higher pregnancy rates [24]. It was shown that measurement of AMH in serum and follicular fluid is a useful prognostic indicator for the outcome of an assisted reproduction attempt. Hattori in 2013 examined the AMH levels in serum and follicular fluid as predictive markers in 58 women, excluding women with polycystic ovary syndrome [25]. Higher clinical pregnancy rates occurred in women who had high levels of AMH in the follicular fluid or serum. They concluded that high AMH levels in either serum or follicular fluid appears to be positively associated with the clinical prognosis of pregnancy. In 2013, Lin et al. reported that AMH may serve as a predictive indicator of the number of oocytes and the good quality of embryos, particularly concerning the percentages of blastocyst formation [26]. It has also been observed that there is a positive correlation between AMH serum level and the number of mature oocytes, the number of oocytes fertilized, and the embryos that developed, in women who reached pregnancy and women who did not [27]. In a study by Capkin et al., AMH was measured in serum and follicular fluid on the day of ovulation. There was a statistically significant, positive correlation between the concentration of AMH in the serum and the total number of oocytes collected. Also, it was observed that both AMH concentration in serum and follicular fluid was higher in pregnant women. Finally, the concentration of AMH in serum and follicular fluid had a statistically significant, positive correlation with implantation rates [28].

As for FSH receptor, there are studies in literature investigating the role of AMH and AMH receptor (AMHRII) gene polymorphisms and the response to IVF protocols. These polymorphisms were associated with IVF parameters: basal FSH levels were lower, fertilization rate was statistically higher, the number of follicles was higher, and total dose of gonadotropins was lower among noncarriers of AMHRII polymorphisms [29]. Recently, the combined study of the most frequent polymorphism of FSHR (Ser680Ans) and AMH type II receptor (-482 > G) genes showed that women that carry one polymorphism have on average 5.5 units higher levels of AMH compared to women carrying no polymorphism. In women with no polymorphisms, for each unit of FSH increase, the average concentration of blood AMH is expected to be 72% lower [30].

The purpose of this study is to examine the FSH receptor Ser680Asn polymorphism in combination with AMH levels in both serum and follicular fluid, on the day of oocyte collection.

The key questions to be answered are:

- Is there a difference between serum AMH (sAMH) value and FSH receptor genotype (Ser/Ser, Ser/Asn, Asn/Asn)?
- Is there a difference between AMH in the follicular fluid (ffAMH) and the FSH receptor genotype (Ser/Ser, Ser/Asn, Asn/Asn)?
- Is there a difference between AMH serum and AMH follicular fluid?
- Since the women in the study will receive different treatment protocols for ovarian stimulation, are there differences in serum AMH and follicular fluid AMH levels between the women in the two protocols?

## 2. Materials and Methods

In this pilot study, a total of 32 women who underwent IVF/ICSI in the 1^st^ Department of Obstetrics/Gynecology of “ALEXANDRA” Maternity Hospital–Division of Reproductive Medicine, Athens Medical School, were included. Inclusion criteria were women 22–42 years of age with no uterine or ovarian anomalies, having normal hormonal profile according to WHO guidelines, a regular menstrual cycle of 21–35 days and both ovaries intact. Each patient underwent a short GnRH-agonist protocol. None of these women had been subjected to ovarian stimulation or any other hormonal treatment for at least three months before entering controlled ovarian stimulation (COS). The protocol was approved by our Ethics Committee, and an informed consent was provided by all participants, according to the Helsinki Declaration.

The following clinical, biochemical, and genetic parameters were recorded: age, years of infertility, number of previous attempts, weight, height, BMI, FSH, LH, AMH serum, AMH follicular fluid, PRL, E2, number of days of stimulation, number of follicles, number of oocytes, maturation rate, number of embryos, fertilization rate, embryo quality, and genotype for the polymorphism Ser680Asn of FSHR.

### 2.1. COS Protocol

A short GnRH-agonist protocol was used: GnRH-agonist buserelin (Suprefact, Sanofi-Aventis) was started on cycle day 2 at a dose of 0.5 mg and was kept until triggering of final oocyte maturation with hCG. HMG (Menopur, Ferring) (21 women) or rFSH (Gonal F, Merck) (11 women) was administered on day 2 at a dose of 200 IU, and the dose was adjusted according to ovarian response on a daily basis. Serum E2 levels were measured daily starting on day 5 of ovarian stimulation with gonadotropins (day 7 of cycle) until the day of triggering final oocyte maturation with 10,000 IU of hCG (Pregnyl, Merck Sharp & Dohme) given intramuscularly. Follicular monitoring started on day 6 of stimulation (day 8 of cycle), and subsequent ultrasound scans were performed every day until oocyte retrieval. Follicular aspiration and oocyte retrieval took place 36 h after the administration of 10,000 IU hCG by transvaginal ultrasound-guided puncture. Luteal phase support was provided with 200 mg of micronized progesterone administered intravaginally three times daily from the day after egg collection onwards, and serum hCG was measured 14 days later. Ultrasound scans, oocyte retrievals, and embryo-transfers were conducted by two fertility specialists of the centre. Similarly, oocyte grading, fertilization, early embryo development, and embryo grading were conducted by two senior embryologists of the centre.

### 2.2. Collection of Peripheral Blood and Follicular Fluid

Hormonal assessments were all performed in the same laboratory. Serum FSH, LH, and PRL was performed on day 3 of the cycle by electro chemiluminescence immunoassay (Roche Molecular, Biochemicals, Mannheim, Germany). The estradiol levels (E2) were measured on day 5 of the cycle of controlled ovarian stimulation followed by every day measurement, until the day of hCG administration, by using a specific kit immunoassay with electrochemiluminescence (chemiluminescent microparticle immunoassay—CMIA kit, Abbott Laboratories, Abbott Park, IL, USA). Serum AMH was measured on day 3 of the cycle as well and also in the follicular fluid on the day of oocyte retrieval, using the immunoenzymatic method ELISA (AnshLabs, Webster, United States).

On the day of ovum pick up, peripheral blood was collected from the patients to perform the genotyping, using an EDTA vial. The samples were stored at -20°C. DNA isolation was performed using the kit “pureLink Genomic DNA kit Invitrogen, USA,” following the manufacturer's instructions. Real-time polymerase chain reaction (PCR) followed, for the detection of Ser680Asn polymorphism, using the device “LightCycler 480 real-time PCR System”-Roche Applied Science. The primers used were FSH-RS AGTGTGGCTGCTATGAAATGC[S] 196599-619 (56.6°C) and FSH-R A GGCTAAATGACTTAGAGGGACAAGTA[A] 196750-729 (56.9°C). The probe used was SP [A] CCCAGAGTCACCAATGGTXITCCA – PH [S] 196697-718 (62.1°C). The protocol is described elsewhere [31].

### 2.3. Statistical Analysis

Statistical analysis was performed with the Statistics Package for Social Sciences (SPSS), version 24, while the Sasieni algorithm (1997) and Hardy-Weinberg equilibrium were performed with the online calculator which is available at http://ihg.gsf.de. The statistical methods used for the control of statistical hypothesis were two independent samples *t*-test, 2 proportion test (normal approximation), and parametric one-way analysis of variance (ANOVA). For qualitative data, the chi-square test was used (Fisher exact test when necessary). The nonparametric tests Mann-Whitney *U* and Kruskal-Wallis test were used when needed. A *p* value of less than 0.05 was regarded as statistically significant. All values are presented as mean ± SD, unless otherwise stated.

## 3. Results

The study included 32 women who were enrolled in an IVF/ICSI program. Two types of gonadotropins were used for ovulation induction: recombinant, human FSH (rFSH, 11 women), and human menopausal gonadotropin (hMG, 21 women). The Ser680Asn polymorphism of the FSH receptor gene and AMH values in both serum and follicular fluid of women were also investigated.

### 3.1. Ser680Asn Genotyping

The distribution of women according to the genotype for Ser680Asn is as follows: 5/32 (15.6%) women were homozygous for the Ser allele (Ser/Ser), 20/32 (62.5%) women were heterozygous for the polymorphism Ser/Asn, and 7/32 (21.9%) women were homozygous for the Asn allele (Asn/Asn). Although the number of participants in this study was small, the distribution of the three genotypes concurs with the prevalence of previous publications in the Greek population.

### 3.2. Clinical and Biochemical Characteristics according to the Ser680Asn Polymorphism

The clinical and biochemical characteristics of the 32 women were recorded. Women were grouped into categories, according to the Ser680Asn polymorphism genotype (as shown in Table 1). As shown in Table 1, no statistically significant difference was observed among women with different genotypes in the following characteristics: age, years of infertility, serum AMH, follicular AMH, LH, estradiol on hCG day, and number of follicles and oocytes. Women homozygous for the Ser allele had higher FSH levels (9.14 ± 2.35 mIU/l) compared to the other two genotypes (*p* value 0.019). Also, the number of mature MII oocytes was lower in Asn/Asn women (4 ± 2), as well as the number of fertilized eggs (4 ± 2) in a statistically significant way (*p* value 0.035 and 0.026, respectively). Also, fertilization rate was higher in Ser/Asn women (0.75 ± 0.15, *p* value 0.021).

Concerning the purpose of the study, no statistical significant difference was found between serum AMH and follicular fluid AMH with FSH receptor genotype for the Ser680Asn polymorphism. Regarding the sAMH/ffAMH ratio in the 3 genotypes, the value was lower in Asn/Asn women (0.37 ± 0.47) than Ser/Ser (0.55 ± 0.35) and Ser/Asn (0.53 ± 0.29), but no statistical significant difference was obtained (*p* value 0.096).

Grouping patients based on whether or not they have the Ser allele we have two groups: homozygous for normal (Ser/Ser) and heterozygous (Ser/Asn) compared to homozygous for polymorphism (Asn/Asn).  Table 2 presents the results; only values that are statistically significant are shown. Women who carry the Ser allele have a higher number of follicles, retrieved oocytes, and mature oocytes than women who do not contain the Ser allele, and this difference is statistically significant for all three parameters.

### 3.3. Clinical and Biochemical Characteristics according to AMH

Women were grouped into categories, according to serum AMH levels. According to Fleming's classification [32], women were classified into two groups. The cut-off point was 2.22 ng/ml. Thus, the first group included women with AMH values < 2.22 ng/ml, labeled as poor responders, while the second group included women with AMH values > 2.22 ng/ml.

The two groups were compared for multiple parameters: age, years of infertility, previous attempts, BMI, FSH, follicular fluid AMH, serum AMH/follicular fluid AMH ratio, LH, estradiol on hCG administration day, number of follicles, number of oocytes, maturation rate, number of fertilized oocytes, and fertilization rate (Table 3).

The two groups were found to have statistically significant differences in age, follicular fluid AMH, and serum AMH/follicular fluid AMH ratio. More specifically, women with serum AMH values < 2.22 ng/ml were older (*p* value 0.015 using *t*-test and *p* value 0.019 using Mann-Whitney *U*), as expected. Also, women with AMH < 2.22 ng/ml presented lower AMH follicular fluid levels (*p* value 0.000 using *t*-test and *p* value 0.001 using Mann-Whitney *U*) and lower serum AMH/follicular fluid AMH ratio in a statistically significant manner (*p* value 0.027 using *t*-test and *p* value 0.041 using Mann-Whitney *U*). Concerning the genotype for the polymorphism Ser680Asn of FSHR in relation to AMH levels, no statistically significant differences were found.

**Figure 1:**
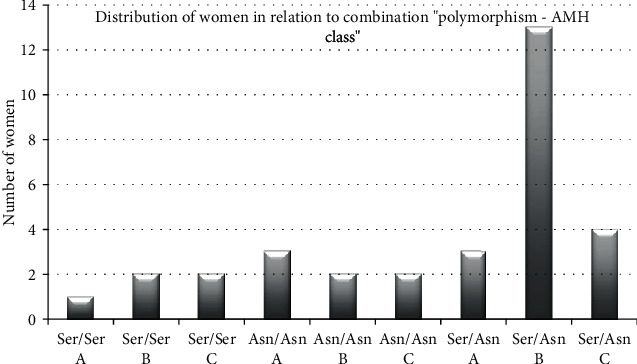
Distribution of women in relation to the combination “polymorphism-AMH class” (*p* value 0.399). Class A corresponds to menopause-related AMH values (0-0.44 ng/ml), class B corresponds to subfertility AMH values (0.44-2.22 ng/ml), and class C symbol corresponds to normal AMH values (2.22 ng/ml).

Figure 1 presents the distribution of women who participated in the study regarding the type of genotype they carry (Ser/Ser, Ser/Asn, Asn/Asn) and the AMH serum levels. Three AMH classes were defined: class A concerns women with serum AMH values corresponding to menopause (0-0.44 ng/ml), class B concerns women with AMH values corresponding to subfertility (0.44-2.22 ng/ml), and class C concerns women with AMH values corresponding to normal fertility (AMH ≥ 2.22 ng/ml). Due to the limited number of women, no further analysis of the traits of each of the categories concerning the ovarian response was done.

The majority of women was heterozygous for the polymorphism Ser680Asn and had AMH values corresponding to subfertility. The combination that had the lowest distribution was the Ser/Ser with AMH values corresponding to menopause.

### 3.4. Clinical and Biochemical Characteristics according to Gonadotropins Treatment

Women were grouped into categories, according to the gonadotropin regimens which were used for ovulation induction. One group included those who received recombinant human FSH (rFSH) and the other group those who received human postmenopausal gonadotropin (hMG).

The two groups of women were found to have a statistically significant difference in the following parameters: number of follicles, number of retrieved oocytes, number of mature oocytes, number of fertilized oocytes, fertilization rate, and number of good quality embryos (Table 4). More specifically, women who received rFSH treatment presented a better profile concerning all of the above characteristics, in a statistically significant way, in both parametric and nonparametric tests.

Subsequently, they were grouped into three categories: women with AMH levels corresponding to menopause (AMH values: 0-0.44 ng/ml), women with AMH levels corresponding to subfertility (AMH values: 0.44–2.22 ng/ml), and women with AMH levels corresponding to fertility (AMH values: ≥2.22 ng/ml). This grouping resulted in a statistically significant difference and is reported in Table 5.

From the serum AMH values of women, it appears that for rFSH treatment (*N* = 11), most women had AMH values corresponding to infertility (81.8%) while the rest had AMH values corresponding to fertility (18.2%). On the other hand, the majority of women who received hMG also had AMH values corresponding to infertility (38.1%), while 33.3% were women with AMH values corresponding to menopause and 28.6% were women with AMH values corresponding to fertility.

## 4. Discussion

The present pilot study was designed to examine the possible combined role of the polymorphism Ser680Asn of FSH receptor and of AMH levels in serum and in follicular fluid, concerning the ovarian response outcome, in women participating in an IVF/ICSI program. It is the first time that the relation of serum AMH and follicular fluid AMH values is evaluated and each of these parameters with the gene polymorphism Ser680Asn of FSHR in women that enrolled in an IVF program. Furthermore, the possible differences in serum AMH and follicular fluid AMH levels between women who received different treatment protocols were investigated.

The identification and determination of the genetic profile of women could be used as a useful tool for predicting the outcome in ART. The future perspective is to individualize the ovarian stimulation and in this direction to personalize the treatment according to each woman's genetic profile. Several studies have focused on the study of gene polymorphism Ser680Asn of FSH receptor, because FSH is a hormone which is necessary for the development and maturation of follicles and acts via its receptor, the granulosa cells of the developing follicle. This particular polymorphism has been associated in many studies with parameters of ovarian stimulation in IVF. The ovarian response to FSH stimulation has been shown to be related to the FSHR genotype [8], and therefore, not only mutations but also polymorphisms in the FSH receptor gene could affect reproductive capacity. Previous studies reported that knowledge of FSHR gene polymorphisms along with other polymorphisms of hormonal receptors could provide information on the effect of ovarian stimulation and the choice of the appropriate protocol [31, 33, 34].

The majority of the study population was heterozygous Ser/Asn (62.5%) for the Ser680Asn polymorphism of the FSHR gene, while the women carrying the Ser/Ser genotype were a minority (15.6%). This frequency is similar to the frequencies described in contemporary literature [8, 31, 33, 35]. Furthermore, the frequencies of these genotypes in the global population according to the meta-analysis of Tang et al. [36] are 40.1% Asn/Asn, 44.6% Asn/Ser, and 16.8% Ser/Ser. In another Greek study, the distribution in the IVF group was Ser/Ser 24.6%, Ser/Asn 59.8%, and Asn/Asn 24.60% [30]. These data indicate that the frequency distribution Ser/Asn in this study is in accordance with the known distribution in literature and this strengthens this study, even if the number of subjects is low.

Another interesting finding is the FSH levels of the study population. Women with Ser/Ser genotype were found to have statistically significant higher levels of FSH, compared to the other two genotypes. This result is in agreement with other researchers [8, 12, 13]. In the cases that are characterized by higher basal FSH serum concentrations, higher amounts of administered FSH are required [37].

This study also showed that the number of mature oocytes was lower in Asn/Asn women as well as the number of fertilized eggs, in a statistically significant manner. Also, fertilization rate was higher in heterozygotes for polymorphism in women. It is worth noting that the number of follicles and oocytes was lower in Asn/Asn women, with a difference that tended to be statistically significant. Thus, a recommendation to start the ovulation induction protocol with higher doses of gonadotropins seems reasonable in the cases that carry the genotypes Ser/Ser or Asn/Asn.

The results of the present study show that the population of women participating in IVF is favored by the presence of the Ser allele. Specifically, women with the Ser allele had a higher number of follicles, oocytes, and mature MII oocytes than women without the Ser allele, and this difference was statistically significant for all three parameters. This finding is consistent with an earlier study [33], in which a greater number of follicles and oocytes were collected in women who were homozygous for the Ser allele.

Furthermore, we analyzed the distribution of women who participated in the study in relation to the combination “ polymorphism-AMH class “, of the 3 genotype and three AMH classes—class A for AMH levels corresponding to menopause, class B for AMH levels corresponding to subfertility, and class C for women with AMH values corresponding to normal fertility. The majority of women was heterozygous for the polymorphism Ser680Asn and had AMH levels corresponding to subfertility. On the other hand, Paschalidou recently studied the -29 (G > A) promoter polymorphism of the FSHR gene, in Greek women undergoing IVF/ICSI. The polymorphic allele for the -29 (G > A) promoter polymorphism correlated with increased number and better quality of oocytes as well. Thus, this observation showed that different polymorphisms of the same gene have different results; thus, we need further studies to identify the appropriate genotype of the phenotypically group of patients that belong to the “subfertility group” or poor responders before IVF attempt, in order to implement special protocols for ovulation induction.

The primary objective of this study was to study the comparison of the serum AMH and of the follicular fluid AMH with the different genotypes of Ser680Asn polymorphism of FSHR. It was observed that AMH levels in both serum and follicular fluid were similar in all three genotypes. Regarding the sAMH/ff AMH ratio in the 3 genotypes, it was observed that the value was lower in Asn/Asn women than in Ser/Ser and Ser/Asn women, but no statistical significant difference was obtained (*p* value 0.096).

Studies have also reported the use of serum AMH for women initiating IVF, not only as a predictor of pregnancy rate [38] but also as a useful tool to personalize treatment in women undergoing IVF fertilization [39]. The results of the present study are in agreement with current literature research on the association of AMH with the age of women. Our findings showed that women with serum AMH levels < 2.22 ng/ml were older than women with serum AMH levels > 2.22 ng/ml in a statistical significant way, as expected.

Another interesting finding of the present study was the statistically significant, positive relation between serum AMH and AMH in the follicular fluid. More specifically, women with serum AMH values < 2.22 ng/ml (AMH levels corresponding to poor responders) had lower AMH levels in the follicular fluid than women with serum AMH levels > 2.22 ng/ml. In addition, it was observed that women with serum AMH levels corresponding to poor responders had lower sAMH/ff AMH ratio compared with the other group of the women, again in a statistically significant manner. Measurement of AMH in follicular fluid presents great practical difficulties compared to serum measurement, and therefore, there are not many studies on it. This observation is supported by earlier studies [25, 28].

In the present study regarding the genetic profile of FSHR, the heterozygotes group presented the lower level of FSH, the higher number of mature MII oocytes and of fertilized eggs, and fertilization rates as well. Many researchers shed light to other polymorphisms, particularly AMH and AMHRII. These results above are in accordance with our previous study on AMH and AMH type II receptor (AMHRII) single-nucleotide polymorphisms (SNPs) Ile49Ser and -482A > G where basal FSH levels were lower, fertilization rate was statistically higher, the number of follicles was higher, and the total dose of gonadotropins was among noncarriers of AMHRII polymorphism [29]. Taking all these under consideration, one could assume that these genetic observations in different SNPs support the notion that the combination of certain genes as AMH and AMHRII polymorphisms, or ESR1,2 polymorphisms, along with the FSHR SNPs in association with parameters of controlled ovarian stimulation may provide means for the prediction of ovarian response in specific subgroups of women entering an IVF/ICSI program. The effort could be even more tempting if the target is the identification of women of poor prognosis.

Regarding the type of gonadotropins that were used for ovulation induction, recombinant or urine, we observed that women that were treated with rFSH predominated in the number of follicles, the number of oocytes, the number of mature MII oocytes, the number of fertilized oocytes, the fertilization rate, the number of good quality embryo, and the thickness of the endometrium. Several systematic studies in the current literature have investigated comparing rFSH and hMG, in *in vitro* fertilization protocols, with the obtained results remaining controversial [40–42]. In the present study, it appears that women treated with rFSH had AMH values corresponding to “infertility” (81.8%) while the rest had AMH values corresponding to “fertility” (18.2%). It is interesting that even if the 81.8% of rFSH group had low AMH value, they showed very good response to ovulation induction regarding the number of follicles, the number of oocytes, and fertilization rate. Nevertheless, we must also underline that none of the women in the rFSH group had very low AMH. This alone is a bias for our conclusions, because this may be the reason why the women that received rFSH presented better outcome (more oocytes, etc.), while women that received hMG had worst AMH values and thus worst outcome concerning the ovarian stimulation protocol.

Day 3 serum FSH, indeed, is considered to be a predictive marker for ovarian function. Also, higher serum AMH levels are associated with great number of retrieved oocytes, and lower serum levels of AMH can predict poor ovarian response. The question that rises is if AMH and/or FSH are reliable for the prediction of outcome of the ovulation induction protocol? Recently, the study of polymorphisms on the receptors of those two hormones in combination, FSHR (Ser680Ans) and AMHR II (-482 > G), showed that women with one polymorphic allele have on average 5.5 units higher levels of AMH compared to women carrying no polymorphic allele. In women with no polymorphisms, for each unit of FSH increase, the average concentration of blood AMH is expected to be 72% lower [30]. However, if we get ahead of this observation, then FSH and AMH values and their gene receptor polymorphisms could be both used as biomarkers for projection of ovarian stimulation outcome.

## 5. Conclusions

The design of this study highlights the possible role of certain markers in the follicular fluid and offers an interesting point of view regarding the value of AMH for the evaluation of the ovarian outcome. The main problem is the small number of the sample; it is obvious that similar questions for the role of SNPs along with markers such as AMH need to be confirmed by other groups of researchers and in larger samples in order to gain more statistical power.

The ideal goal in the future would be to use the appropriate genetic markers to better predict the ovarian response to ovulation induction protocol and offer better results to our patients. The identification of polymorphisms, such as Ser680Asn of FSHR in this study, along with the determination of endocrine markers in the follicular fluid, such as AMH, could lead at some point, to the personalized therapy setting per woman, based on DNA sequencing. Particularly useful is the science of biostatistics, which uses algorithms in information programs, and enables simultaneous and rapid analysis of multiple gene loci and their association with the factors involved in follicular development, implantation, and pregnancy (genome-wide association studies—WGAS).

## Figures and Tables

**Table 1:** Clinical and biochemical characteristics of women of three groups based on the genotype for the polymorphism Ser680Asn of FSH-R. Values in bold are statistically significant (*p* value ≤ 0.05). ^∗^One-way analysis of variance (Kruskal Wallis).

Parameters	Ser/Ser (*N* = 5)	Asn/Asn (*N* = 7)	Ser/Asn (*N* = 20)	*p* value^∗^
Age	33 ± 4	34 ± 2	35 ± 4	0.394
Years of infertility	2 ± 1	4 ± 3	4 ± 3	0.076
FSH (mIU/l)	9.14 ± 2.35	7.72 ± 2.24	6.13 ± 1.37	**0.019**
Serum AMH (ng/ml)	2.95 ± 3.85	1.05 ± 0.99	1.28 ± 1.08	0.477
Follicular AMH (ng/ml)	3.94 ± 3.00	3.58 ± 1.75	2.56 ± 1.78	0.186
sAMH/ffAMH (ng/ml)	0.55 ± 0.35	0.37 ± 0.47	0.53 ± 0.29	0.096
LH (mIU/l)	5.39 ± 1.36	4.63 ± 1.91	5.31 ± 2.14	0.562
E2 on day of hCG (pg/ml)	2869.60 ± 2119.40	1437.57 ± 683.54	2249.55 ± 921.54	0.152
Number of follicles	8 ± 2	6 ± 3	9 ± 2	0.094
Number of oocytes	8 ± 2	6 ± 2	8 ± 2	0.09
Number of mature oocytes	6 ± 2	4 ± 2	7 ± 2	**0.035**
Number of fertilized oocytes	5 ± 2	4 ± 2	6 ± 2	**0.026**
Fertilization rate	0.62 ± 0.13	0.64 ± 0.12	0.75 ± 0.15	**0.021**

**Table 2:** Comparison of clinical profiles of women based on whether or not they carry the Ser allele in their genotype. ^∗^Mann-Whitney *U*.

Parameters	Ser/Ser and Ser/Asn (*N* = 25)	Asn/Asn (*N* = 7)	*p* value^∗^
Number of follicles	8.84 ± 1.91	6.43 ± 2.64	**0.032**
Number of oocytes	8.20 ± 1.94	5.86 ± 2.54	**0.028**
Number of mature oocytes	6.56 ± 1.94	4.29 ± 1.70	**0.01**

**Table 3:** Clinical and biochemical profiles of women with AMH values < 2.22 ng/ml (poor responders) and women with AMH values > 2.22 ng/ml that underwent IVF. ^∗^*t*-test, ^∗∗^Mann-Whitney *U*.

Parameters	♀ with AMH < 2.22 ng/ml (*N* = 24)	♀ with AMH > 2.22 ng/ml (*N* = 8)	*p* value
Age	35 ± 3	32 ± 3	**0.015** ^∗^ **0.019** ^∗∗^
Years of infertility	4 ± 3	3 ± 2	0.417^∗^ 0.598^∗∗^
Previous attempts	1 ± 1	1 ± 1	0.876^∗^ 0.180^∗∗^
BMI	23 ± 3	21 ± 3	0.228^∗^ 0.317^∗∗^
FSH (mIU/l)	7.0 ± 2.1	6.8 ± 1.9	0.863^∗^ 0.931^∗∗^
Follicular AMH (ng/ml)	2.1 ± 1.1	5.5 ± 2.0	**0.000** ^∗^ **0.001** ^∗∗^
sAMH/ffAMH (ng/ml)	0.4 ± 0.3	0.7 ± 0.4	**0.027** ^∗^ **0.041** ^∗∗^
LH (mIU/l)	5.2 ± 2.1	5.0 ± 1.5	0.777^∗^ 0.879^∗∗^
E2 on day of hCG (pg/ml)	2006.4 ± 991.9	2656.1 ± 1606.9	0.183^∗^ 0.361^∗∗^
Number of follicles	8 ± 2	9 ± 2	0.661^∗^ 0.724^∗∗^
Number of oocytes	8 ± 2	8 ± 2	0.659^∗^ 0.808^∗∗^
Number of mature oocytes	6 ± 2	6 ± 2	0.924^∗^ 0.912^∗∗^
Maturation rate	0.8 ± 0.1	0.7 ± 0.1	0.256^∗^ 0.293^∗∗^
Number of fertilized oocytes	6 ± 2	5 ± 2	0.716^∗^ 0.826^∗∗^
Fertilization rate	0.7 ± 0.1	0.6 ± 0.2	0.198^∗^ 0.228^∗∗^

**Table 4:** Clinical and biochemical characteristics of women according to the gonadotropin regimens. For all data in the table, *p* values were calculated with both parametric (*t*-test—^∗^) and nonparametric tests (Mann-Whitney *U*—^∗∗^).

Parameters	Women under rFSH treatment (*N* = 11)	Women under hMG treatment (*N* = 21)	*p* value
Age	34 ± 4	35 ± 4	0.436^∗^ 0.472^∗∗^
Years of infertility	4 ± 3	4 ± 2	0.708^∗^ 0.888^∗∗^
Previous efforts	1 ± 2	1 ± 1	0.683^∗^ 0.908^∗∗^
BMI	21 ± 3	23 ± 4	0.225^∗^ 0.222^∗∗^
FSH (mIU/l)	6.7 ± 1.3	7.1 ± 2.3	0.508^∗^ 0.874^∗∗^
Serum AMH (ng/ml)	1.3 ± 0.8	1.6 ± 2.2	0.649^∗^ 0.321^∗∗^
Follicular AMH (ng/ml)	2.5 ± 1.5	3.2 ± 2.2	0.321^∗^ 0.487^∗∗^
sAMH/ffAMH (ng/ml)	0.6 ± 0.3	0.4 ± 0.3	0.306^∗^ 0.123^∗∗^
LH (mIU/l)	5.4 ± 2.4	5.1 ± 1.8	0.658^∗^ 0.827^∗∗^
E2 on day of hCG (pg/ml)	2113.4 ± 975.6	2197.9 ± 1297.4	0.851^∗^ 0.890^∗∗^
Number of follicles	10 ± 1	7 ± 2	0.000^∗^ 0.002^∗∗^
Number of oocytes	9 ± 1	7 ± 2	0.003^∗^ 0.003^∗∗^
Number of mature oocytes	7 ± 1	5 ± 2	0.009^∗^ 0.014^∗∗^
Maturation rate	0.8 ± 0.1	0.8 ± 0.1	0.733^∗^ 0.842^∗∗^
Number of fertilized oocytes	7 ± 2	5 ± 2	0.00^∗^ 0.002^∗∗^
Fertilization rate	0.8 ± 0.1	0.7 ± 0.1	0.065^∗^ 0.083^∗∗^
Number of good embryos	6 ± 2	3 ± 3	0.021^∗^ 0.053^∗∗^

**Table 5:** Comparative table of different treatments based on AMH values of the study population. The *p* value was calculated by the Fisher's exact test.

Serum AMH (ng/ml)	rFSH (*N* = 11)	hMG (*N* = 21)
0-0.44	0 (0%)	7 (33.3%)
0.44-2.22	9 (81.8%)	8 (38.1%)
≥2.22	2 (18.2%)	6 (28.6%)
*p* value = 0.036		

## Data Availability

The data of women used to support the findings of this study are available from the corresponding author upon request.
